# A Qualitative Comparative Study of the Doctor-Patient Relationship Model and Decision-Making Style among Chinese and Japanese Doctors

**DOI:** 10.1007/s41649-025-00389-7

**Published:** 2026-05-05

**Authors:** Hua Xu, Taketoshi Okita, Masao Tabata, Atsushi Asai

**Affiliations:** 1 Department of Medical Ethics, Tohoku University Graduate School of Medicine, Sendai, Miyagi Prefecture Japan; 2 Division of Philosophy and Ethics, Department of Culture and Medicine, School of Medicine, Shiga University of Medical Science, Otsu, Japan; 3 Patient Safety Management Office, Tohoku University Hospital, Sendai, Japan; 4 Department of Medical Ethics, Tohoku University Graduate School of Medicine, Sendai, Japan

**Keywords:** Qualitative descriptive analysis,, Cross-cultural comparison,, Doctor-patient relationship,, Decision-making style,, China,, Japan

## Abstract

The nature of doctor-patient relationships (DPRs) and decision-making style (DMS) is related to patient satisfaction, clinical outcomes, and the occurrence of disputes. Given the importance of understanding the current state of DPRs, we conducted an exploratory analysis of the current state of DPRs and DMS in clinical settings in China and Japan. We explored similarities and differences between the two countries, as well as influential factors in DPRs and DMS. Twenty doctors in China and 20 doctors in Japan were recruited through the authors’ personal networks as well as snowball sampling, and semi-structured interviews were conducted. Doctors were asked about mainstream DPR models and DMS in current medical practice, and their responses were analyzed using qualitative descriptive methods. Results of the analysis suggest that, in both China and Japan, a diverse mosaic of DPRs and DMS coexists. In both countries, there are paternalistic relationships between doctors and patients, as well as teacher-and-student relationships, but there are also cases in which patients make final decisions and those which adopt shared decision-making. Some doctors referred to DPRs as a friendship. Furthermore, attitudes of individual doctors and patients, the medical environment, trust between the two parties, and national and cultural characteristics strongly impact the formation of DPRs. Our findings highlight the importance of carefully monitoring the current state and various influencing factors of DPRs in order to realize effective human relationships in clinical settings.

## Background

Various doctor-patient relationship (DPR) model classifications have been presented in the literature from social, legal, and decision-making role perspectives (Asai et al. 2021; Hoffmann et al. 2022; Veatch 1972). A summary of DPR models, major decision-making styles (DMS), and representative direct metaphors is provided in Table 1 (Asai et al. 2022; Ahuja 2019; Childress and Childress 2020; Emanuel and Emanuel 1992; Entwistle and Watt 2016; Veatch 1972; Shah et al. 2025; Yonemura 2023). In recent years, the deliberative model, which is suitable for realizing shared decision-making (SDM) between patients and doctors, has become the preferred model (Ahuja 2019; Veatch 1972).

**Table 1:** Classification of major doctor-patient relationship (DPR) and decision-making style (DMS)

DPR model		Explanation	Doctors’ roles (simile)
Paternalistic model (beneficence model)		“*Doctors decide which treatment is best and talk to the patient only for their consent, and in extreme cases, just inform the patient of the intervention to be carried out*.” (Emanuel and Emanuel 1992; Ahuja 2019)	Parent Priest Guardian
Patient-informed choice (consumerist or informative model)		*Doctors provide information about treatment options but patients decide their own treatment*.” (Emanuel and Emanuel 1992; Ahuja 2019)	Technician Engineer Provider
SDM Narrow SDM (interpretative model) Broad SDM (collegial model or deliberative model)		*Doctors assist patients in determining which treatment is most in line with their values*.” (Emanuel and Emanuel 1992; Ahuja 2019)	Facilitator Coach Counselor Adviser
		“*Doctors engage the patient in a dialogue to help determine and choose the best health related values and achieve the best outcome that can be achieved in a patient’s specific situation*.” (Emanuel and Emanuel 1992 and Ahuja 2019)	Teacher Friend Colleague
Contractual model		*“The content of medical care is decided through free negotiation between doctors and patients who are on an equal footing.”* (Higuchi 1998; Yonemura 2023)	Equal individual
Fiduciary relationship model		*“It is based on the premise of asymmetry between doctors and patients, and the fiduciary relation in which doctors have a duty to act solely in the interests of their patients.”* (Higuchi 1998; Yonemura 2023)	Trustee

Arguably, the ultimate goals of medicine can only be achieved when good doctors practice good medicine and when all those participating in medical care are satisfied (Grundnig et al. 2022). Much research has been published and normative discussions held on the characteristics that good doctors possess (Bhugra and Ventriglio 2024; Schnelle and Jones 2023; Stergiopoulos and Martimianakis 2023). In many countries, patients want doctors who are welcoming, focused, empowering, respectful, humane, humble, unprejudiced, trustworthy, empathetic, thorough, and personal. These attributes are universally accepted as desirable attributes of a good doctor (Wang et al. 2023). The question “What should a doctor do?” is almost never asked in isolation; rather, the real question is “What should the doctor do in this context and relationship?” (Boyd et al. 1997). Therefore, “good doctor”-related issues are important when considering the characteristics of an ideal DPR. There are also ongoing debates about the pros and cons of religious beliefs, ideal professional boundaries, and the possibility of true friendship between doctors and patients (Asai et al. 2021; Ruiz-Moral 2022; Silk 2024). Doctors and patients may have different priorities when it comes to characteristics that are needed in doctors in DPRs. One study suggests that, compared to patients, doctors tend to focus less on interpersonal aspects such as care, appreciation, and empathy (Berger et al. 2020; Baro Vila et al. 2023). Thus, there is a need to continuously examine critically and constructively the ethics of DPRs.

While there is a wealth of discussion on the topic of the ideal model of DPRs and characteristics of a good doctor, the DPR model that is predominant in modern clinical settings has not been touched on from a descriptive perspective. The nature of DPRs and DMS is related to patient satisfaction, clinical outcomes, and the occurrence of disputes, and it is important to understand the current state of these and explore relevant factors that influence DPRs (Xu et al. 2024). DPRs can have a substantial impact on healing and/or harming (Boyd et al.^1997^), and they can facilitate doctors’ knowledge of patients, patient trust in doctors, and management (Habermann-Horstmeier and Horstmeier 2023; Tsatsani et al. 2024). Thus, gaining a better understanding of the relationship between doctors and patients in clinical settings is an urgent issue. Indeed, none of the conceptualizations in the literature are likely to capture the complexity and variation existing in actual encounters between patients and doctors (Entwistle and Watt 2016).

Against this backdrop, the overall objective of the present qualitative study is to clarify the current state of DPRs in clinical settings in China and Japan. Specifically, we aim to investigate what kind of DPR models exist in the two countries; reveal whether a novel DPR model other than the models described in Table 1 exists; identify similarities and differences between DPRs in the two countries; analyze factors that affect the DPRs models and DMS; and attempt to reveal doctors’ impression on the current state of DPRs as well as DMS in clinical settings.

In this regard, China and Japan have similar cultures but differing healthcare systems. We believe that comparative research of China and Japan is informative for several reasons (Ahuja 2019; Hiraga et al. 2020; Xu et al. 2024, 2025). First, such research can demonstrate how differences in cultural and social aspects, interpersonal differences, and historical changes in the two countries are related to the current state of DPRs and DMS in each country. Second, experiences and perceptions of doctors from both countries can provide useful information for people from both countries to learn about and from each other. Third, future opportunities to access each other’s medical systems from citizen-level exchanges between China and Japan would likely benefit from insight gained from such comparisons. Accordingly, we sought to deepen our understanding by comparing the current state of medical care in both countries.

## Methods

**Fig. 1:**
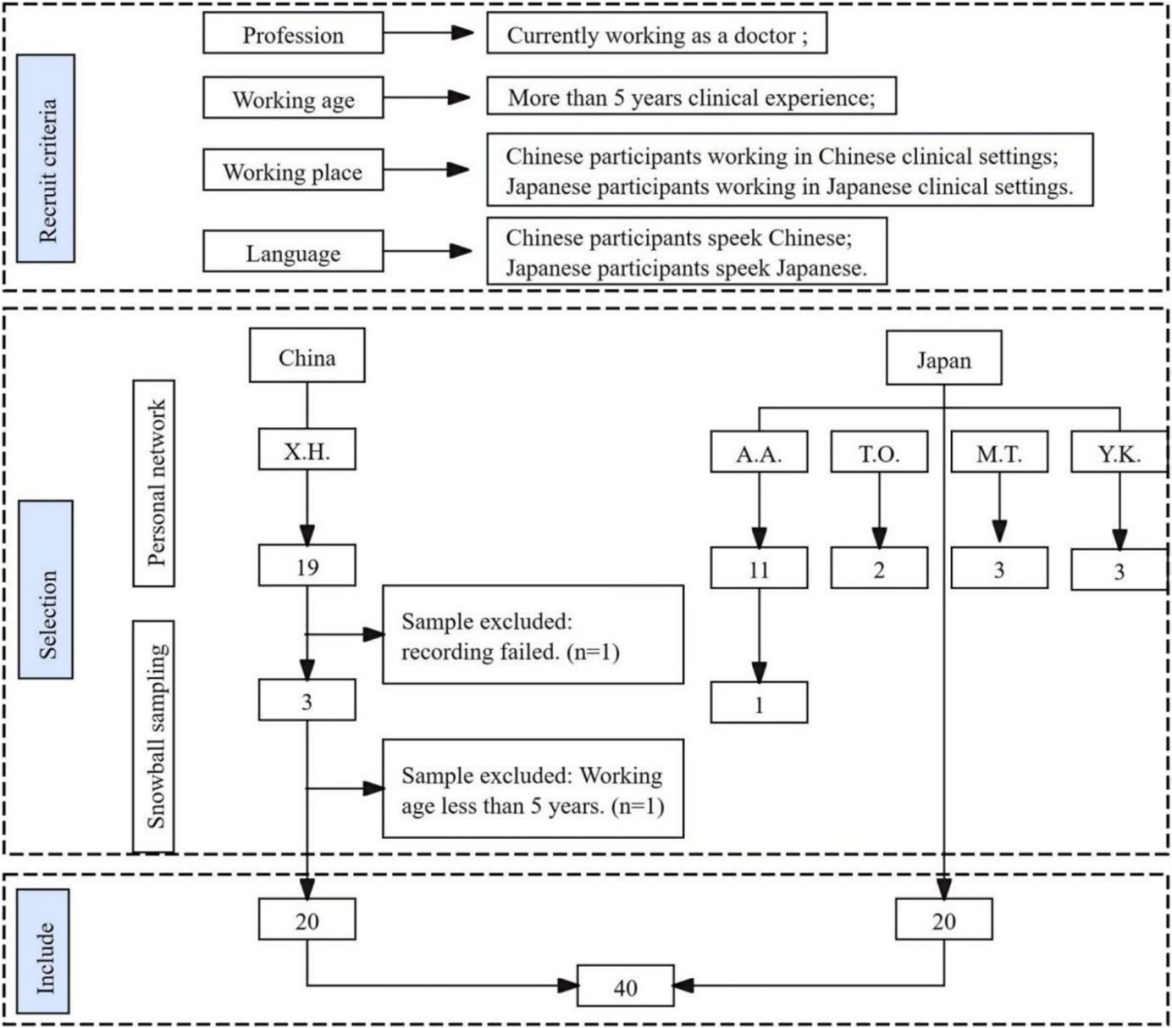
Forty participants recruitment flowchart

The present study is part of a China-Japan comparative study on DPRs, doctor-patient disputes, and doctor well-being. At present, we have published two papers, “Reflections from Chinese and Japanese Physicians on Medical Disputes” and “Thoughts and behaviors of Chinese and Japanese doctors when faced with the death of a patient: a qualitative descriptive study of doctors’ responses to a hypothetical scenario,” based on the findings of the study so far in the *Asian Bioethics Review*, and the present paper is the third one from the same study. Table 2 shows the brief summary of our three papers. In the main study, semi-structured interviews were conducted with 20 Chinese doctors and 20 Japanese doctors regarding DPR, DMS, the current state of medical disputes, and doctor well-being in both countries, using the same questions. Doctors in both countries were sampled through the researchers’ personal networks. Snowball sampling was also used for some participants. All participating doctors were currently working doctors who had already completed their clinical training by the time of the interviews (Fig. 1).

**Table 2:** Summary of our three articles

Title	Reflections from Chinese and Japanese Physicians on Medical Disputes (Xu et al. 2024)	Thoughts and behaviors of Chinese and Japanese doctors when faced with the death of a patient: a qualitative descriptive study of doctors’ responses to a hypothetical scenario (Xu et al. 2025)	A qualitative comparative study of the doctor-patient relationship model and decision-making style among Chinese and Japanese doctors (the present paper)
Research aims	Doctor-patient conflicts, workplace violence, and direct involvement in disputes have a significant negative impact on the well-being of physicians and doctor-patient relationship (DPR). The study aimed to compare the experiences and perceptions of Chinese and Japanese physicians regarding medical disputes	In clinical settings, admissions of medical error and apologies by doctors continue to be an important but difficult issue. The study aimed to compare the thoughts and behaviors of Chinese and Japanese doctors when faced with the unexpected death of a patient	While there is a wealth of discussion on the topic of the ideal model of DPRs and characteristics of a good doctor, the DPR model that is predominant in modern clinical settings has not been touched on from a descriptive perspective. The overall objective of the present qualitative study is to clarify the current state of DPRs in clinical settings in China and Japan. Specifically, we aim to investigate what kind of DPR models exist in the two countries; reveal whether a novel DPR model other than the models described in Table 1 exists; identify similarities and differences between DPRs in the two countries; analyze factors that affect the DPRs models and DMS; and attempt to reveal doctors’ impression on the current state of DPRs as well as DMS in clinical settings
Research method	Qualitative descriptive case series analysis based on participating doctor’s statements	Qualitative descriptive analysis of participating doctors’ responses to a hypothetical scenario involving the death of a young man	Qualitative descriptive analysis of participating doctors’ answers concerning predominant DPR and DMS in current clinical settings
Main research results	Common issues in medical disputes include monetary motives of patients and/or families, violence/threats from patients and/or families, the inability of patients and/or families to understand the risk of complications, and the uncertainties of medicine. Additionally, medical disputes have serious impact for the well‑being, careers, and professionalism of physicians. As for the differences in the two countries, strong distrust exists before seeing a doctor in patients and/or their families and concentration of patients in large hospitals cause complicated disputes in China; however, inadequate explanation by physicians of treatment plans or potential complications to patients and/or their families causes disputes in Japan	There were some disagreements concerning responsibility for the patient’s death, communication with the bereaved family, disclosure of medical errors, and apology. Doctors from both countries stated that the fight (the patient’s action in the scenario) itself was responsible for the patient’s death; however, some doctors predicted that the doctor and hospital would lose the lawsuit. Many Chinese doctors noted that it is impossible for doctors to communicate with the family due to the possibility of physical violence from bereaved family members; however, several Japanese doctors suggested that the first major premise is to properly explain what happened to the family. In China, the magnitude of the medical error changes doctors’ attitudes towards honest disclosure; however, some Japanese doctors thought that if there was a medical error, it should be explained to the family as soon as possible	A total of seven DPR models were identified including paternalism, consumerism, friend-like relationship, teacher-student relationship, contractual relationship, consultant-client relationship, and shared decision-making (SDM). Coexistence of different relationship models as well as internal and external factors influencing the development of DPRs and DMS in China and Japan were pointed. Attitudes of individual doctors and patients, the medical environment, trust between the doctors and patients, and national and cultural characteristics concerning human relationships strongly impact the formation of DPRs
Research insights	In order to avoid unnecessary conflicts, contradictions, and distrust between doctors and patients, it is not enough to work only at the level of the medical system and hospital management. Healthcare should be maintained as much as possible as a public good, and it is important to reduce the financial burden on patients and their families in order to prevent disputes arising from money-related reasons. Doctors should consider building moral trust to gain interpersonal trust when interacting with patients. Neither patient violence nor inappropriate media coverage can be eliminated by the efforts of doctors alone. It requires raising the level of morality in society as a whole. The most important point of morality here is reciprocity	Educating doctors about the ethics of medical error disclosure and the importance of apologizing is likely to be insufficient to generate a true apology, particularly so unless the present healthcare system successfully eliminates barriers to apologizing, such as violence against doctors upon disclosing errors and apologies in China. Die-hard concern that admitting the occurrence of adverse events and showing condolence and regret can be perceived almost equal to accepting responsibility in both countries must also be resolved. It is of great importance to realize a clinical environment where conscientious doctors need not hesitate to admit medical errors and can willingly apologize to those who have been harmed	Our results implied that current situations in both China and Japan are inconsistent with the alleged historical development of the normative ethics of DPRs and DMS, which idealizes SDM. It can be argued that there is no single perfect DPR model, and each model has its merits and drawbacks. The uniqueness of the individual relationship may be the justification for the co-existence of different DPRs. Our findings also highlight the importance of carefully monitoring the current state and various influencing factors of DPRs in order to realize effective human relationships in clinical settings. Given the significant impact of DPRs on outcomes and patient satisfaction, further research is urgently needed

Interviews were conducted in both countries over a 2-month period spanning March and April 2023. This study was approved by the Ethics Committee of Tohoku University Graduate School of Medicine on January 26, 2023 (2022–1-886). At the time of conducting the interviews, the method and intent of the study were explained, and all participants provided written informed consent and were assured that the contents of the interviews would be recorded and that their statements would be reported anonymously. No honorarium was paid for participation in the study.

Qualitative descriptive analysis was used to develop subcategories and categories concerning DPRs in both countries (Alshahrani et al. 2022; Gibbs 2018; Miles and Huberman 2018). Our qualitative description followed the philosophical foundations of natural inquiry, usually describing a participant’s experience directly or presenting an event in simple language as free of artifice as possible in the artifice-laden enterprise known as conducting research (Lindgren et al. 2020; Ma et al. 2023; Sandelowski 2000).

The initial steps of all analyses were conducted by Hua Xu (H.X.) and Atsushi Asai (A.A.). H.X. and A.A. then shared their results with the other researchers (T.O., M.T). Finally, discussions were held with all researchers until a consensus was reached on the contents. The steps taken to analyze the data are described in the following. First, each audio recording file (either in Chinese or Japanese) was transcribed into a Word document and translated into English after each interview. H.X. and A.A. read the verbatim transcripts several times to obtain a complete picture of the interviewees’ responses. Second, meaning units related to the research themes in 40 Word documents were listed in two Excel files (one each for Chinese and Japanese doctors), followed by coding of the meaning units in each file. Third, codes with similar content were grouped into subcategories. The subcategories correspond to the DPR model in the following. We continued the same process iteratively, and all researchers confirmed and agreed on the appropriateness of all generated codes and subcategories. “CD” means Chinese doctor, while Japanese doctor is represented by “JD.” Relevant portions of the opinions of doctors regarding DPR are also provided here in the form of direct quotes. During the analysis, all researchers, including four (H.X, A.A, T.O, and M.T) with experience conducting qualitative research, engaged in discussions to ensure the consistency, validity, and reliability of data.

Prior to asking our interviewees about DPRs and DMS, we presented them with examples of DPR models that mainly use direct metaphors and the three main DMS (Table 1) to encourage them to express their own opinions. We then asked them the questions: “What is the mainstream doctor-patient relationship in the current medical field?” and “What is the mainstream decision-making model in the current medical field?” Below, we present results of the inductive analysis of the answers to these two questions. Notably, we did not perform a deductive analysis using existing DPRs/DMS (Table 1) as preexisting codes. Given the above background, DPRs and DMS were occasionally expressed by participants using similar and overlapping terms, and the concepts of DPRs and DMS were used interchangeably. Therefore, results of the analyses relating to decision-making and relationships are described together.

## Results

The background of participating doctors is shown in Table 3. A total of seven DPR models were identified as in Table 4. All DPRs were referred by doctors from both countries. Coexistence of different relationship models as well as internal and external factors influencing DPRs and DMS in China and Japan were pointed out by our participants. Subcategories mainly showing DPR models are underlined, and quotes are italicized.

**Table 3:** Demographic characteristic of the study participants

Category	China	Japan
	No. (%) or mean ± SD (***N*** = 20)	No. (%) or mean ± SD (***N*** = 20)
Interview time mean (min)	50 (27–148)	52 (37–80)
Gender		
Female	4 (20)	3 (15)
Male	16 (80)	17 (85)
Age		
20’s	1 (5)	0
30’s	4 (20)	1 (5)
40’s	12 (60)	5 (25)
≧50’s	3 (15)	14 (70)
Working experience	16 ± 8	29 ± 13
5–9 y	4 (20)	0
10–19 y	12 (60)	2 (10)
20–29 y	2 (10)	9 (45)
≧30 y	2 (10)	9 (45)
Hospital type		
Primary healthcare institution		5 (25)
Secondary hospital		6 (30)
University hospital	20 (100)	9 (45)
Specialty		
Internal medicine	2 (10)	14 (70)
ER	3 (15)	
Surgery	14 (70)	6 (30)
ICU	1 (5)	
Conflict experience with patient and/or its family		
Aggressive attitudes/words	19 (95)	2 (10)
Physical attack	6 (30)	1 (5)
Personal/professional involvement in formal dispute process	17 (85)	7 (35)

**Table 4:** Doctor-patient relationship mentioned in the study participants’ interviews

	**Model**
1	Paternalism
2	Friend-like relationship
3	Teacher-student relationship (quasi-paternalism)
4	Contractual relationship
5	Technician-consumer relationship (patient-informed choice model)
6	Consultant-client relationship (quasi-paternalism)
7	Shared decision-making (deliberative model)

### Paternalism in China and Japan

Chinese doctors expressed the view that, at present, DPRs and DMS tend to be paternalistic. First, during the decision-making process, doctors possess professional knowledge and skills that patients do not, leading patients to rely on and comply with the advice of doctors. Second, greater patient compliance often results in better outcomes, encouraging doctors to make decisions on behalf of their patients, thereby placing the final decision-making authority with doctors. However, there is a discrepancy between ideal medical decision-making and reality. According to medical law, patients have the right to be informed about the details of their treatments and make their own decisions, and doctors are responsible for providing adequate information and explanations to this end. However, in clinical practice, doctors often make the decisions for patients.

“*I believe the decision-making process aligns with paternalism. In practice, we often make decisions for patients. While patients have the right to be informed, doctors possess the professional expertise. Therefore, decision-making should be primarily guided by the doctor. Most of the time, patients are compliant and can understand the rationale behind the decisions*.” [CD4].

On this topic, Japanese doctors expressed the view that the clinical environment has shifted from a paternalistic model to a patient-informed choice model in the past 30 years. As a result, doctors are now more gentle and less arrogant than in the past. However, deep within the doctors, the paternalistic mindset has not changed much, with the outward display being different from their actual mindset. That is, while Japanese doctors act like an equal partner, they actually consider patients to be just like children. Therefore, in clinical practice, doctors still play a paternal role and inevitably maintain a dominant position in DPRs, building a hierarchical relationship with their patients. While the decision-making process appears to follow a patient-informed choice model or shared decision-making model on the surface, the reality is that DPRs align more closely with a paternalistic approach. There are several reasons for this. First, Japanese patients often find it difficult to make decisions independently and usually defer to doctors. Some senior doctors limit the options presented to patients, which can lead to conflicts in DPRs later on. Second, while doctors who provide patients with extensive information seemingly adhere to the patient-informed choice model, actual communication between patients and doctors remains predominantly unilateral because patients tend to feel obliged to follow doctors’ suggestions. As a result, doctors tend to take the lead, and patients are not genuinely free to make their own choices. Third, doctors often believe their decisions are in the best interest of patients. Once an agreement is reached regarding treatment, the expectation is that patients will follow their guidance. Higher patient compliance is associated with better treatment outcomes and more harmonious DPRs.

“*The father-child relationship is common when a patient undergoes a major surgery like transplantation. In this case, I would explain things to them just like a father does to his child*.” [JD15].

### Friend-Like Relationship in China and Japan

DPRs typically reflect an equal relationship between adults. However, when patients have consulted the same doctors for a long period, their relationship may take on the form of a friendship.

“*To me, when the patient visits, it’s more like seeing one of my friends for the first time in a while*.” [CD20].

In Japan, there is friendship between doctors and patients, without any hierarchy. Japanese doctors expressed the view that they can learn much from patients, not only in terms of accumulating medical experience, but also learning about different lifestyles, values, and ways of thinking, and that it would be even better if patients could be more proactive in the decision-making.

“*In my outpatient clinic, we’re friends. Patients are those who you’ve known for a long time, so we talk almost as if we’re friends*.” [JD1].

### Teacher-Student Model in China and Japan

One Chinese doctor expressed the view that, in current clinical settings in China, the DPR adopts a teacher-student model.

“*During the diagnosis and treatment process, most of time, the DPR adopts more of a teacher-student model*.” [CD11].

This sentiment was shared by some Japanese doctors as well. In clinical settings, doctors should provide various options and take the lead, as they have more knowledge and experience. In particular, when it comes to home care, doctors have the final responsibility for patient care and must make decisions that enhance the quality of life of patients, knowing that there is a certain risk that adverse events could occur (e.g., bathing for the elderly at high risk of cardiac arrest).

“*In the eyes of my patients, I am more like a respectable person, like a monk or a school teacher, than a parent or child*.” [JD13].

### Contractual Model in China and Japan

Some Chinese doctors expressed the view that DPRs are just a professional or contractual relationship.

“*Not a friend. Merely a relationship between a doctor and patient, I mean, a contractual relationship*.” [CD4].

More than half of the Japanese doctors expressed the view that their relationship with patients was a contractual one, i.e., a contract between a doctor and patient. In other words, DPRs are based on mutual interests, with both parties having certain responsibilities and obligations. They share common goals, and patients can legitimately expect some benefits from doctors.

“*It’s important to create an atmosphere similar to that of a teacher and student in a cram school, where patients can ask questions openly and doctors try to provide information in an easily understandable way. In DPRs, there should be a contractual agreement and due expectation*.” [JD17].

### Technician-Consumer Model in China and Japan

Some Chinese doctors expressed the view that DPRs take on a consumerist model. Although referring to DPRs as a technician-consumer relationship seemingly violates medical ethics, which regards the shared decision-making (SDM) model as ideal, in essence, DPRs are a consumerist relationship. Decision-making is typically based on the patient-informed choice model, which is also a consumerist model. Doctors provide treatment options and plans based on their professional knowledge and offer explanations to patients, and the ultimate decision-making authority lies with the patient.

“*I think the relationship between doctors and patients is like a consumerist relationship. The patient is like a boss, and the doctors should serve the boss*.” [CD13].

“*We provide multiple treatment options and explain the pros and cons of each to patients. However, doctors are only advisers and not the final decision-makers*.” [CD1].

Some Japanese doctors expressed the view that DPRs are a businessman-consumer relationship. Doctors are technical personnel, and patients are consumers. Doctors spend time meeting the demands of patients and are required to provide them with information about treatment options and explanations, allowing patients to make their own decisions. However, true informed consent arguably is not fully implemented in the decision-making in current DPRs. This is because patients frequently make decisions without fully understanding treatment plans, leading them to choose alternatives which may not be in their best interest. In such cases, doctors can only comply with patients’ decisions since the ultimate decision-making authority rests with patients due to the strong emphasis on patient autonomy.

“*Generally speaking, the DPR is the kind of relationship seen between technical personnel and consumers. For example, many patients simply want medication or temporary relief from pain*.” [JP18].

“*The days of paternalism are over. Current clinical practice places too much emphasis on respecting the patients’ wishes. Doctors have no choice but to approve patients’ decisions because it is the patients’ will. This is the opposite of the paternalistic model, and leaves too much room for patients to make the decision*.” [JD9].

### Consultant-Client Relationship in China and Japan

Some Chinese doctors expressed the view that DPRs involve both guiding and being guided. Doctors act as consultants who provide advice to their patients.

“*The DPR involves guiding and being guided. Doctors play the role of advisors, while patients are seen as clients*.” [CD3].

Some Japanese doctors expressed the view that DPRs are an expert-client relationship. Doctors provide guidance based on their medical expertise to patients who have complaints and requests.

“*The DPR is not a completely equal relationship because the relationship includes the provision of professional expertise*.” [JD14].

### Shared Decision-Making (SDM) Model in China and Japan

Some Chinese doctors expressed the view that, in the decision-making process, doctors provide patients with treatment options and explanations, and then both parties make decisions through mutual discussion and collaboration. However, the application of SDM in actual clinical practice remains relatively limited.

“*During the decision-making process, there is mutual collaboration between doctors and patients. Doctors present treatment options, and decisions are ultimately made through negotiation between both parties*.” [CD12].

Some Japanese doctors expressed the view that, despite its importance, SDM remains ideal. First, although promoting equality is a societal trend and present-day healthcare strives to establish patient-centered care, DPRs have not yet achieved equality. Both doctors and patients believe that doctors should be respected, so if doctors try to act as equal partners, both parties may feel at a loss. Second, differences between doctors and patients, in which the former are professionals and the latter are not, can make it difficult for each to reveal their true thoughts. Third, there needs to be an equal relationship between doctors and patients, as this can help prevent medical disputes that may result from overlooking patients’ thoughts.

“*There is a certain difference between doctors and patients resulting in mutual resistance in acting equally in DPRs. For example, it is difficult for a patient to reveal himself to a doctor, or a doctor to reveal himself to a patient*.” [JD10].

Other Japanese doctors expressed the view that the decision-making model tends towards SDM. In DPRs which apply SDM, doctors and patients collaborate, with the fundamental aim of respecting the decisions of patients and their families and doctors assisting in the decision-making process. Younger doctors today are more inclined towards SDM, offering patients more treatment options to choose from.

“*I personally prefer SDM. I explain the advantages and disadvantages of various treatment methods and ensure that the patient fully understands them, allowing the patient to make their own choice. It is crucial to consider factors such as side effects and financial issues when discussing treatment options. Ultimately, treatment begins with the agreement of both parties*.” [JD13].

### Coexistence of Different Relationship Models in China and Japan

Some Chinese doctors expressed the view that, in clinical practice, some DPRs resemble a consumerist relationship, while others resemble a friendship model or a consultant-client relationship. It is also claimed that all modes except paternalism exist in China.

“*In the ENT department, I think DPRs adopt more of a counselor or friendship model, rather than a consumerist one. Many patients have chronic conditions, such as rhinitis and chronic pharyngitis, so they often consult the same doctor privately*.” [CD17].

A Japanese doctor expressed the view that the current mainstream DPR model has features of paternalistic and consumerist models.

“*The paternalistic model and consumerist model are half and half, respectively, in a mosaic*.” [JD6].

### Internal and External Factors Influencing DPRs and DMS in China and Japan

Doctors from both countries commented on factors that influence DPRs and/or DMS. Chinese doctors expressed the view that DPR models are flexible because they are often affected by factors such as the level of trust between doctors and patients, the personality of doctors, the condition of patients, and the medical environment in which DPRs exist. Some doctors noted that the decision-making process in DPRs is shaped by patient background factors, including cognitive ability, economic status, and the nature and severity of the disease. Although doctors fundamentally treat patients equally regardless of their status, the way and methods of explanation may vary depending on the patient’s background or clinical situation.

“*If patients trust doctors, it is the friend model. Otherwise, it would be the consumerist model*.” [CD6].

“*The decision-making model is fundamentally based on trust. Most patients accept treatment advice from doctors because they trust them, enabling collaboration between doctors and patients*.” [CD19].

“F*or elective surgeries, patients have more freedom to choose, while for emergency surgeries, patients have fewer choices, and the doctor determines the treatment plan*.” [CD20].

Similar to Chinese doctors, Japanese doctors expressed the view that DPR models are difficult to fix. On the one hand, DPR models depend on the patient’s background, including age and condition. For example, elderly patients often do not make decisions actively and tend to rely on doctors. On the other hand, DPR models are influenced by factors on the medical side, including the type of medical institution and its geographical location, doctors’ generation (age), and healthcare practices. For example, doctors working in rural medical institutions tend to be paternalistic. In contrast, medical institutions that focus on home care differ significantly in the type of care they provide compared to general hospitals. In medical institutions that focus on home care, DPRs are more like a partnership. Trust is built through interactions with families and patients, creating a supportive environment. Within this environment of trust, the medical team carefully selects the best treatment plan for patients. The utility of SDM is, however, limited in clinical settings where one life-saving treatment is clearly desirable over the alternatives. Japanese doctors also noted that only about 10% of cases manage to achieve SDM and they often lack the time and energy required to implement SDM effectively. Furthermore, doctors frequently overlook medical ethics principles, such as respecting patients’ autonomy and preferences.

“*Insufficient time to implement SDM*.” [JD9].

“*We want to respect the patient’s will and decision, but it is not always easy, because the family’s wishes, the doctor’s wishes, and the patient’s condition do not always match up. In addition, the family’s cooperation is sometimes poor*.” [JD20].

## Discussion

**Fig. 2:**
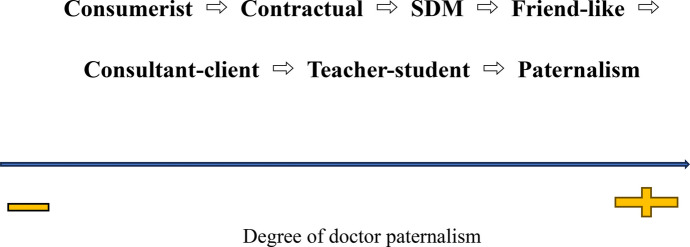
A spectrum of doctor-patient relationships based on the degree of paternalism

This study revealed several aspects of DPRs and DMS in clinical settings in China and Japan. First, diverse models of DPRs coexist in a mosaic-like fashion in both China and Japan; decision-making is carried out in various styles, and the ethical principles which are prioritized differ. The paternalistic model also appeared to be the predominant model in both countries (Fig. 2). Second, there were gaps both between ethical and legal ideals, which recommend guaranteeing a patient’s right to self-determination and SDM, and between the actual situation in China and Japan. There were obstacles to the realization of these ideals at various levels, including the individual, clinical, and medical environmental levels. Third, there was a gap between the superficial attitudes of Japanese doctors towards their patients and their feelings towards decision-making. While doctors may act in accordance with the SDM model on the surface, their true feelings are paternalistic, and this inner tendency gives rise to manipulative communications with patients. Fourth, there were generational and cultural differences that affected DPRs and DMS. The mode of DPR referred to as the “teacher-and-student relationship” in both Japan and China was more similar to the paternalistic model than to the deliberative model or SDM model proposed by Emanuel and Emanuel (1992). Fifth, there was a perception that a friendship was established between doctors and patients in both Japan and China, with long-term DPRs potentially leading to feelings of trust and friendship. Some Chinese doctors noted that, although mutual trust between doctors and patients could change the consumerist relationship into a friendship, the consumerist model can also become a boss-servant relationship in which patients are the boss.

Based on the perspectives of doctors from both countries on DPRs and DMS, we focus the subsequent sections on the following topics: paternalism, the consumerist model, SDM, and similarities and differences between these in China and Japan.

### Paternalism Taken for Granted by Doctors

Doctors in both countries agreed that they are the authority on medicine and knowledge, often leading to a high patient dependence and low autonomy in medical decision-making. Doctors in both countries also indicated that the higher the patient compliance, the better the medical outcomes. Thus, although paternalism is increasingly challenged in contemporary clinical settings, doctors take the practice of paternalism for granted in medical decision-making.

However, we believe that paternalism being the norm in clinical settings is problematic for at least the following reasons. First, that a patient’s dependence and obedience in medical decision-making is usually a passive or forced choice is easily overlooked by doctors. The background of patients, including their personal desires, personalities, and cultural environment, as mentioned by Japanese doctors, together with the various social rules and rules of interpersonal relationships, not to mention having to deal with the diseases, is enough to make patients passive, dependent, and submissive. For example, Japan has five psychocultural-social tendencies—“surmise"(*Sontaku*), “self-restraint” (*Jishuku*), “air” (atmosphere or mood, *Kuuki*), “peer pressure” (or tuning pressure, *Docho-Atsuryoku*), and “community” (*Seken*). And these are considered characteristics of the present-day Japanese population and may negatively affect the ideal practice of SDM in clinical settings (Asai et al. 2022) and increase the opportunity for doctors to implement paternalistic practices.

Second, a patient’s dependence and compliance can change over time. Japanese doctors noted that the era of paternalism will eventually pass. With the development of social economy, information technology, and refinement of the social division of labor, modern interpersonal relationships have been characterized by “increasing autonomy and decreasing dependence”(Guan and Xin 1988). Thus, increased patient autonomy and decreased dependence appear to be contemporary features of modern DPRs.

Third, doctors from both countries indicated that the higher the patient compliance, the better the outcomes. However, to the best of our knowledge, there is no conclusive evidence that higher patient compliance leads to better medical outcomes. Moreover, ethics and laws tend to enrich and strengthen patients’ rights, and the concept of commercialization of healthcare has simultaneously fueled the desire of patients for better (or even perfect) medical treatment. Indeed, according to the literature, there is a tendency to measure the effectiveness of medical outcomes using patient-reported outcomes (PRO) (Hua 2025).

Finally, when practicing paternalism, doctors of the present study always considered the relevant laws and ethics in medical decision-making. In particular, Chinese doctors noted that the conflict between law and ethics is an obstacle to practicing paternalism. For example, paternalism is described in Table 1 as follows: “Doctors decide which treatment is best and talk to the patient only for their consent, and in extreme cases, just inform the patient of the intervention to be carried out.” We often think about what the optimal choice is and how a patient’s true consent can be obtained. However, the law provides a clear—albeit controversial—measure for determining what is right, wrong, and best. Through informed consent, the law grants patients the right to make decisions and imposes on doctors the duty to disclose relevant information. In this manner, laws resolve the question of what constitutes the “best choice” and how to obtain consent, although whether it is reasonable or not is a topic for further discussion. In both China and Japan, there are countless cases of doctors being held liable in court for the failure to properly obtain informed consent (Hui et al. 2024; Masaki et al. 2014; Ohira 2023), making doctors who adopt paternalism feel wronged and forced to admit their negligence.

DPRs and the dominant role of doctors in medical decision-making have largely changed historically (Childress and Childress 2020). The modern era has introduced new dynamics into DPRs, characterized by increasing patient autonomy and a growing need for a legal framework to regulate and guide the behaviors and ethics of both doctors and patients. As a result, the ethical foundations of paternalism face mounting challenges, and the consumerist model has emerged in response to these challenges.

### The Controversial Consumerist Model (Patient-Informed Choice Model)

The consumerist model (Table 1) is present in both countries. Both Chinese and Japanese doctors indicated that under this model, doctors should strictly adhere to full information disclosure and fully respect patient autonomy. However, attitudes toward this model were not very positive in both countries due to various factors that are detrimental to DPRs.

First, Chinese doctors indicated that the consumerist model violates interpersonal trust and medical ethics and hinders SDM. They noted the near complete lack of trust between doctors and patients with this model. The consumerist model is also perceived to be at odds with medical professional ethics. Chinese doctors often exhibit certain behaviors that are commonly regarded by their peers or patients as unethical, including accepting bonuses, taking drug rebates, prescribing excessively, engaging in overtreatment, and accepting “red envelopes” (monetary gifts from patients). Such practices are frequently cited as the main reasons for the stigmatization of Chinese doctors (Wang et al. 2019). However, these unethical behaviors in the “Chinese-style” consumerist model are essentially absent in current Japanese clinical settings. This may have to do with improvements in the Japanese healthcare system, drug management system, legal regulatory system, and other systems (Xu et al. 2024). The consumerist model as perceived by Japanese doctors takes on a different form—patients with a consumer mindset often are simply seeking medication or temporary relief.

Second, in the consumerist model, DPRs extend far beyond a simple buyer–seller dynamic, placing broader and more demanding expectations by patients on doctors. In addition to the difficulty of meeting patient expectations, as Japanese doctors noted, doctors face the challenge of accepting the reality that their authoritative status and respected image in the paternalistic model have been replaced, as noted by Chinese doctors. In the consumerist model, we argue that healthcare services are viewed as something akin to commodities. The importance of healthcare quality is undeniable for both patients as consumers and medical institutions as providers of healthcare services. The WHO evaluates healthcare quality using indicators that include treatment outcomes, patient safety, and patient experience (such as costs, adequate consultation time, clear explanations, opportunities to ask questions, and participation in decision-making). To improve healthcare quality, doctors naturally must bear the burden of greater pressure. Respecting patient rights, considering medical costs, ensuring patient safety, and improving treatment outcomes require doctors to have more effective communication skills, more accurate risk assessment abilities, advanced professional expertise, and higher ethical qualities, which are often difficult to achieve consistently.

Third, the lack of or failure to obtain informed consent is increasingly becoming an issue in the adjudication of medical dispute cases. Doctors perceived that the consumerist model is nearly identical to patient-informed choice (informative model) (Table 1). While correlations between paternalism, the consumerist model, and the incidence of disputes related to inadequate informed consent remain unclear, cases arising from the failure to obtain informed consent are on the rise in China (Masaki et al. 2014; Zhang et al. 2025).

In summary, the introduction of the consumerist model into DPRs, together with the various issues it raises, is often regarded as a “deviation from ethics.” In the consumerist model, doctors generally hope to mitigate their medical responsibility by fulfilling their duty of informed consent. However, their responsibilities have not diminished; rather, the pressures of medical practice have intensified.

### SDM, the Seemingly Ideal Model

Compared to the paternalistic and consumerist models, SDM has been more readily accepted by doctors from both countries. However, doctors also noted that SDM has been achieved only in limited cases, and its realization takes time.

First, to achieve SDM, Chinese and Japanese doctors indicated that DPRs need a longer duration to be more effective. Japanese doctors also stressed the importance of DPRs to undergo a qualitative transformation, whereby both doctors and patients adapt to and redefine their roles in medical decision-making. This role transformation and adaptation aim to bring both parties closer to equal standing. As noted by Japanese doctors, such role shifts would be extraordinarily challenging for both doctors and patients and cannot be accomplished in a short period. For example, a situation in which a father suddenly becomes a friend to his child or a student immediately treating a teacher as a peer on their first meeting could be challenging and even inconceivable.

Second, cultural background plays a crucial role in practicing SDM. Cultural differences between the East and the West influence the interpretation and acceptance of SDM. Our interviews suggest that, in Japan and China, the teacher-student relationship is usually categorized as more of a paternalistic model than SDM. This categorization sharply differs from that suggested by Emanuel and Emanuel in 1992. In the East, traditional culture assigns a “teacher” the same status as that of a parent. “Once a teacher, always a father (Chen and Wang 2019).” “To treat your teacher is like treating your parent” (Jin 1998). Teachers should be treated with the same respect as a father, and a father’s order cannot be resisted. Therefore, it would be difficult for patients in both China and Japan to accept teachers and parents as friends or colleagues, and patients may experience guilt in doing so.

Third, according to Japanese doctors, unavoidable inequality in DPRs, such as disparities in professional knowledge and status, pose significant barriers to the adoption of SDM. However, China’s situation differs from Japan’s, and many of the factors that deepen doctor-patient inequalities are human-induced. In China, issues such as high medical costs, the prevalence of “red envelopes,” and incidents of violence against healthcare workers have distorted DPRs and deepened the inequality between doctors and patients (Li et al. 2025). Furthermore, the degree and nature of doctor-patient inequality vary across countries due to differences in clinical environments, and this also influences the implementation of SDM. Thus, the achievement of SDM appears to be a long way off in both countries. Maintaining long-term stability and equality can facilitate role transitions and adaptations, which is conducive to the practice of SDM. Additionally, differences in characteristics of both traditional culture and healthcare background unique to each country also significantly influence the practical application of SDM.

### Limitations

The present study has several limitations. First, the examples provided in Table 1 may have influenced the respondents’ comments and could have prevented them from discussing DPRs and DMS in their own words. If we had simply asked what the mainstream DPR in current clinical settings is like without providing specific examples, respondents may have raised completely new metaphors and/or models. Second, although the present study was qualitative and exploratory in nature, only limited generalizations can be made based on our results. Our results cannot be generalized to other countries and are far from conclusive, even when considering only the two targeted countries. Indeed, the state of DPRs described here may be specific to only the Far East, and DPRs in other countries may take on a completely different form.

Third, differences in background between physicians from both countries (e.g., hospital type and age) may also have affected the validity of our comparisons. We cannot rule out the possibility that differences in the types of medical institutions where our participants in China and Japan worked, their specialties, and the proportion of male and female participants (there were more male participants than female participants) may have influenced the results of the present study. However, due to the exploratory qualitative nature of this study, it is difficult to discuss statistical correlations between respondents and their responses. Compared to qualitative research, quantitative research has stricter standards regarding sampling methods and bias prevention (Li 2017a; Li 2017b; Pyo et al. 2023). On the other hand, we primarily relied on our personal networks to recruit participants for specific purposes and did not perform random sampling or sample size calculations beforehand to control for bias and error. We also did not establish any hypotheses to be verified in advance.

Based on the findings of this study, however, there appear to be no significant differences in opinions between male and female respondents. In the chronic care setting where the doctor-patient relationship is long-term, friendship may have developed between doctors and patients, or a more equal relationship may have been established, compared to the acute care setting. Regarding the frequency of DPR models, Chinese participants mentioned consumerism more frequently than Japanese participants. All DPR models, as well as DMS, were mentioned by doctors from both countries.

Furthermore, according to recent review articles, it is unclear how the gender of doctors influences their decision-making processes (Champagne-Langabeer and Hedges 2021). Meanwhile, a recent study from Dubai suggested that female doctors, compared to their male counterparts, are more engaged in SDM with both male and female patients (Alameddine et al. 2022). Finally, to the best of our knowledge, there is no evidence that a physician’s specialty influences DPRs or DMS, at least in the case of physicians in China and Japan. However, a Japanese doctor argued that acute care is highly urgent, and paternalism on the part of doctors is acceptable, with patients tending to play a passive role; on the other hand, in chronic care, treatment decisions are largely left to the autonomy of patients who play an active role (Ito 2023).

There may also be regional differences within a country. Fourth, even within the same culture, individuals significantly differ in many ways. The differences in attitudes and ideas of the doctors may have resulted from differences in individual upbringing, life experiences, and discipline at home. Furthermore, different generations of doctors may have different attitudes and behavior patterns. Individuals can also significantly change their cultural perspectives as life progresses. Within the same culture, some aspects change over time, while others remain constant (Masaki et al. 2014). Fifth, this study only explores the DPR model and DMS from the perspective of doctors, and no patient perspective is included. We will provide our discussion in this regard in the conclusion to address the importance of patient centeredness. Finally, interviews were conducted in Chinese or Japanese, and then analyzed and presented in English. This process could have introduced translation issues, such as misunderstanding of the details and/or true meaning of the physicians’ comments. However, given the fluency of some of the researchers in Chinese, Japanese, and English, we were able to confirm the quality and authenticity of the English translation (Xu et al. 2024).

## Conclusion

The results of the present study suggest that a variety of DPRs, including paternalism and consumerism, exist in clinical settings in China and Japan. This is inconsistent with the historical development of the normative ethics of DPRs, which idealizes SDM. Given the significant impact of DPRs on outcomes and patient satisfaction, further research is needed on this topic on a global scale. It will also be necessary to consider what disadvantages or benefits the existence of diverse DPRs have on patient care. The results of the present study raise many questions. Is the coexistence of diverse DPRs ethically problematic? What are the reasons for the mosaic of DPRs, and does this coexistence cause chaos in medical practice, or, conversely, does the chaos in medical practice give rise to the mosaic? Should we aim to standardize only the most ideal DPR, and is practicing the ideal DPR feasible in current clinical settings? Or would it be possible only in the future? In addition, being a “good doctor” is thought to be a necessary condition for establishing good DPRs; however, is being a good doctor sufficient for good DPRs? What are good DPRs in the first place?

The present study is a qualitative descriptive study conducted in doctors with the aim of clarifying the current state of DPRs and DMS from the perspective of doctors working in modern China and Japan. There are numerous empirical studies on DPRs that focus solely on doctors as research subjects. There is also a trilogy of qualitative studies that presents findings on whole-person care as well as holistic or biopsychosocial care based on the results of interview surveys conducted by the same doctor-researchers (Thomas et al. 2020). We believe that these circumstances suggest that doctors recognize the importance of a good DPR and that their views on this topic are also important. However, given the importance of patient-centered care in recent years, we felt that it is also essential to discuss the DPR and DMS desired by patients themselves and suggest the current state from the patients’ perspective in the present paper.

It is stated, “Patient-centeredness focuses on the patient’s experience of illness and health care and on the systems that work or fail to work to meet individual patients’ needs. Similar terms are *person-centered, consumer-centered, personalized, and individualized*. Like these terms, patient-centered encompasses qualities of compassion, empathy, and responsiveness to the needs, values, and expressed preferences of the individual patient.” (Gerteis et al. 1993) identified several dimensions of patient-centered care: (1) respect for patients’ values, preferences, and expressed needs; (2) coordination and integration of care; (3) information, communication, and education; (4) physical comfort; (5) emotional support—relieving fear and anxiety; and (6) involvement of family and friends (Gerteis et al. 1993). In addition, it is argued that patient-centered communication incorporates the patient’s perspective and promotes partnership between doctors and patients (Susilo et al. 2023).

The important question then is which DPR achieves patient-centered care. According to the definitions and principles found in the literature, it would be the SDM model (Ahuja 2019; Childress and Childress 2020; Susilo et al. 2023). The friend-like or contract model mentioned by our respondents may also achieve patient-centered care by definition; however, it is important to be mindful of how personal emotions may come into play in the friend-like model, and problems with SDM should also be taken into account, including psychological burden on patients arising from decision-making participation, unstable and negative emotional state, various cognitive biases, and cultural tendencies such as obedience to authority, familism, and heteronomy rather than autonomy (Asai et al. 2021, 2022). As mentioned in the Introduction section, in many countries, patients want doctors who are welcoming, focused, empowering, respectful, humane, humble, unprejudiced, trustworthy, empathetic, thorough, and personal. These attributes are universally accepted as desirable attributes of a good doctor (Wang et al. 2023). Doctors with these attitudes may be able to provide patient-centered care.

However, different patients may have different priorities when it comes to doctors’ characteristics that are needed in DPRs and DMSs. Patients’ decision-making and attitudes toward doctors may also change depending on the stage of their diseases (Gans et al. 2023). Previous studies have suggested that, in clinical encounters, patients stressed the importance of being heard by their doctors, being given enough care, adequate time, and attention, developing a friendly relationship, and actively engaging in a SDM process. Communication and trust between patients and doctors are the key to a good relationship (Čartolovni et al. 2023; Grundnig et al. 2022). One recent interview study revealed that patients felt being seen as a person when physicians were understanding and caring and, according to the participants, empathy could be expressed by saying “I notice what it does to you, it is intense.” The participants also provided narratives describing how they experienced doctors’ lack of empathy and felt that doctors did not always recognize their unease or behaved inappropriately, which evoked negative emotional reactions (Debets et al. 2024). It was also reported that older men wanted to participate in the medical encounter and be involved in their care, and that their preferred involvement varied along a continuum ranging from “quasi-involvement” to “taking charge,” with most participants being in the middle, preferring a “partnership” patient-physician relationship. The researchers argued that these results differed from previous studies that found that older adults did not tend to want to participate in decision-making (MacRae 2024).

Moreover, based on the current situations of patient participation in SDM, there seems to be room for improvement to achieve better patient-centered care. One study investigated 54 video-recorded surgeon–patient consultations and demonstrated that patient perspectives (ideas, concerns, and expectations) emerged in less than one third of consultations. Surgeons typically addressed patient perspectives when raised, but less frequently initiated discussion on these topics, resulting in the rarity of patient-initiated ideas and expectations. The researchers argued that there is a need for surgeons to actively engage with patient perspectives offered in consultations, emphasizing the importance of respect for patient knowledge and expectations to improve patient satisfaction and healthcare outcomes (White et al. 2024).

A recent qualitative interview study with psychiatric patients also suggested that different forms of decision-making were used, and patients’ expectations and perceptions regarding SDM varied, resulting in the diverse degree of patient involvement with a spectrum ranging from passive to active behavior. The researchers suggested that patients’ families or peer support workers can be involved to strengthen patients’ role in decision-making (Gurtner et al. 2024). A systematic review using a qualitative meta-summary in 2025 also suggested that stigma and self-stigma still exist in mental healthcare and continue to suppress patients’ self-efficacy to participate in SDM (Mertens et al. 2025). Furthermore, a multi-country study concerning patient-reported roles in decision-making conducted in five Asian countries including Bangladesh, China, India, Sri Lanka, and Vietnam suggested that a significant portion (from about 40% to about 60%, depending on the country) of patient respondents with advanced cancer were not involved in decision-making. However, joint decision-making with doctors and/or family members was associated with better quality of life and care. The majority (73%) of respondents participated in decision-making at a level concordant with their preferences, and 15% of them reported that they were involved more than preferred while 11% did less. The researchers recommend doctors to explain the benefits of SDM to their patients while ensuring that patients feel supported and do not find decision-making overwhelming in the situation where collectivistic tendency is culturally predominant (Ozdemir et al. 2021).

Based on the abovementioned findings concerning the current state of patients regarding SDM and influencing factors, what actions should we take now? We should not forget that the individual DPR depends on multiple factors, including human individuality and chance, and there may not be a single DPR that applies uniformly to all patients (Asai et al. 2025). At the same time, however, the ultimate aim of healthcare is, we believe, to alleviate or eliminate patient suffering in both mind and body through effective and humane care. We should pursue a DPR and DMS that enable us to realize the alleviation of human suffering and enhancement of individual well-being, and to this end, it is important for doctors to be flexible and adapt to each patient’s situation (Asai et al. 2025). Doctors should also be sensitive to patient preferences for decision-making participation at the time, as well as barriers that undermine patients’ willingness to participate in decision-making. To achieve these goals, ethical education, medical humanities education, and team-based clinical education are essential to cultivate a spirit of respect for patients among doctors and to enable them to develop the ability to build patient-centered relationships that are in the best interests of patients. Further investigation is needed, with the next step being a large-scale, detailed survey of the current status of DPRs and DMS in patients, physicians, and other healthcare professionals using both quantitative and qualitative methods.

Finally, from an impartial ethical perspective, i.e., a universal perspective, we must search for a good DPR that suits everyone. “Everyone” would include patients and their families, medical professionals including doctors, medical facilities, the general public, and the government. The purpose of medical care is to alleviate or eliminate the suffering of patients, but we must also consider the safety and well-being of medical professionals, continuation of the medical system, maintenance of universal health insurance, and the lives of all citizens when thinking about a good DPR. We must consider the rights and obligations, responsibilities, hopes and intentions, ideals and realities, and the future of healthcare for all stakeholders.

In conclusion, we believe that DPRs must be humane and good in and of themselves. At the same time, they must result in desirable outcomes in patient care. In Utopia, perhaps, a perfectly harmonious DPR could develop in a comfortable setting, with sincere and friendly interactions, mutual trust established, mutual satisfaction as a result, and no disputes or violent confrontations occurring afterwards. Unfortunately, however, such a utopia does not exist. In reality, we must continue to consider various challenges related to DPRs and DMS, as well as aspects of DPRs that are universally, ethically acceptable.

## Data Availability

The datasets used by and/or analyzed during the study are available for the corresponding author upon reasonable request.
